# Testing alternative hypotheses for the decline of cichlid fish in Lake Victoria using fish tooth time series from sediment cores

**DOI:** 10.1098/rsbl.2023.0604

**Published:** 2024-03-20

**Authors:** Nare Ngoepe, Alenya Merz, Leighton King, Giulia Wienhues, Mary A. Kishe, Salome Mwaiko, Pavani Misra, Martin Grosjean, Blake Matthews, Colin Courtney Mustaphi, Oliver Heiri, Andrew Cohen, Willy Tinner, Moritz Muschick, Ole Seehausen

**Affiliations:** ^1^ Aquatic Ecology and Evolution, Institute of Ecology and Evolution, University of Bern, 3012 Bern, Switzerland; ^2^ Department of Fish Ecology and Evolution, EAWAG, Swiss Federal Institute for Aquatic Science and Technology, 6047 Kastanienbaum, Switzerland; ^3^ Institute of Geography and Oeschger Centre for Climate Change Research, University of Bern, Altenbergrain 21, 3013 Bern, Switzerland; ^4^ Institute of Plant Sciences, University of Bern, Altenbergrain 21, 3013 Bern, Switzerland; ^5^ Tanzania Fisheries Research Institute (TAFIRI), Dar es Salaam, Tanzania; ^6^ Geoecology, Department of Environmental Sciences, University of Basel, 4056 Basel, Switzerland; ^7^ Department of Geosciences, University of Arizona, Tucson, AZ, USA; ^8^ Groningen Institute for Evolutionary Life Sciences, University of Groningen, Nijenborgh 7, 9747 AG, Groningen, Netherlands; ^9^ Center for Water Infrastructure and Sustainable Energy (WISE) Futures, Nelson Mandela African Institution of Science and Technology, P.O. Box 9124, Arusha, Tanzania

**Keywords:** haplochromine cichlids, *Lates niloticus*, fish fossils, eutrophication, extinction

## Abstract

Lake Victoria is well known for its high diversity of endemic fish species and provides livelihoods for millions of people. The lake garnered widespread attention during the twentieth century as major environmental and ecological changes modified the fish community with the extinction of approximately 40% of endemic cichlid species by the 1980s. Suggested causal factors include anthropogenic eutrophication, fishing, and introduced non-native species but their relative importance remains unresolved, partly because monitoring data started in the 1970s when changes were already underway. Here, for the first time, we reconstruct two time series, covering the last approximately 200 years, of fish assemblage using fish teeth preserved in lake sediments. Two sediment cores from the Mwanza Gulf of Lake Victoria, were subsampled continuously at an intra-decadal resolution, and teeth were identified to major taxa: Cyprinoidea, Haplochromini, Mochokidae and Oreochromini. None of the fossils could be confidently assigned to non-native Nile perch. Our data show significant decreases in haplochromine and oreochromine cichlid fish abundances that began long before the arrival of Nile perch. Cyprinoids, on the other hand, have generally been increasing. Our study is the first to reconstruct a time series of any fish assemblage in Lake Victoria extending deeper back in time than the past 50 years, helping shed light on the processes underlying Lake Victoria's biodiversity loss.

## 1. Introduction

Ecology and evolutionary biology have long been fascinated with the enormous diversity of cichlid fish in the African Great Lakes. Studies of this endemic diversity have had a major influence on ecological and evolutionary sciences by addressing questions about exceptional species diversity, the evolutionary history of these fish, and the processes at play when diversity is lost, as well as the relevance for ecosystem processes and conservation. The plight of the highly speciose haplochromine cichlids of Lake Victoria (LV) gained further attention in the twentieth century as fish community structure and diversity were drastically modified by major environmental and ecological changes, culminating in the extinction of approximately 200 endemic cichlid species. The diversity of this species flock, which represents the product of the fastest known large-scale speciation episode [1,2], has likely been affected by numerous factors including anthropogenic eutrophication [3], fishing pressures [4] and introduced non-native species [5]. Initial reports suggested the extinction of hundreds of cichlid species was primarily due to predation by Nile perch [5,6]. Subsequent studies suggested that a combination of fishing (especially the targeting of larger cichlids [7]), eutrophication of the lake [8,9], and predation synergistically caused this extinction [10]. The collapse of the cichlid community affected the ecosystem of LV as much as it affected the livelihoods of millions of people, and it is the largest mass extinction ever witnessed by scientists.

The earliest published fishing survey in LV took place in 1927–1928 [11], but the first lake-wide bottom trawl survey was not conducted until 1969 [12]. These early surveys reported broad taxonomic categories of fish and the species-rich haplochromine cichlids as a single group. In Mwanza Gulf, standardized monitoring using trawling began in 1978, a few years before the mass extinction of cichlids which occurred in 1987/88 [13]. These trawl data cannot conclusively inform us about the timing of the onset of the decline of cichlids and the debates about the causes persist [14]. Therefore, a longer record of fish community composition is necessary to test predictions of temporal trends in the composition and abundance of key taxa made by the various hypotheses regarding impacts of fishing, eutrophication, and Nile perch predation and competition.

Palaeolimnological data are a valuable source to infer historical changes in taxon composition and the abundance of fossilizing organism groups associated with past environmental changes [15]. Examining fish remains embedded in lake sediment cores can reveal previously undocumented temporal dynamics in the fish assemblage. Fossil assemblages in African Great Lake sediments commonly contain fish remains, such as teeth [16], that accumulate in the sediments following shedding during tooth replacement throughout the life of a fish and/or upon death with deposition of the carcass. Fish tooth morphology carries information that can be used to address ecological questions [17], and resolve taxonomic compositions [18,19]. Our study analysed fish teeth from sediment cores in Mwanza Gulf, LV, and we reconstructed changes in fish populations over the past two centuries and correlated them with major environmental changes.

## 2. Methods

Two sediment cores (SC9 and SC14) were taken with a UWITEC GmbH (Mondsee, Austria) gravity corer [20] (electronic supplementary material, SI*. 2*) from the Mwanza Gulf of LV, Tanzania (electronic supplementary material, figure S1). The cores were collected at a water depth of approximately 12.5 m and approximately 14.5 m, respectively, and captured the sublittoral part of the Mwanza Gulf [6]. Each core was subsampled continuously at 1 cm intervals (for 28 cm) and the macrofossil analysis was carried out following the methodology described in Ngoepe *et al*. [19] and elaborated further in electronic supplementary material, SI*. 2*. The coring locations were chosen to fall along the established research transect of the Haplochromine Ecology Survey Team and Tanzania Fisheries Research Institute [7]. This is the only part of LV where fish biodiversity and biomass have been measured and monitored at the species level with the same standard methods since before the arrival of Nile perch, albeit at irregular intervals during the past 45 years. The resulting data documented the massive loss of cichlid species and biomass in the mid-1980s [6,21]. Recovered fish teeth fossils were identified and assigned to taxa using a reference collection of teeth from extant species of fish in LV [19] and also published fish tooth photographs, drawings, and descriptions [22–27]. The core chronologies of SC9 and SC14 were based on geochronological data and visual correlation points with hyperspectral TChl profiles from SC12, a core collected from the same location as SC14, from King *et al*. [28] (electronic supplementary material, figure S2, SI. *3*). To identify temporal change points of fossil teeth concentration (teeth per cm^3^), and we used the segmented package v. 1.6-4 in R (regression models with break-points and change-points estimation) [29] and Spearman's correlation to analyse the trends (more details electronic supplementary material, SI. 4; SI. *5*) (Mochokidae was excluded as only one tooth fossil was found electronic supplementary material, SI. *6*).

## 3. Results and discussion

**Figure 1.**
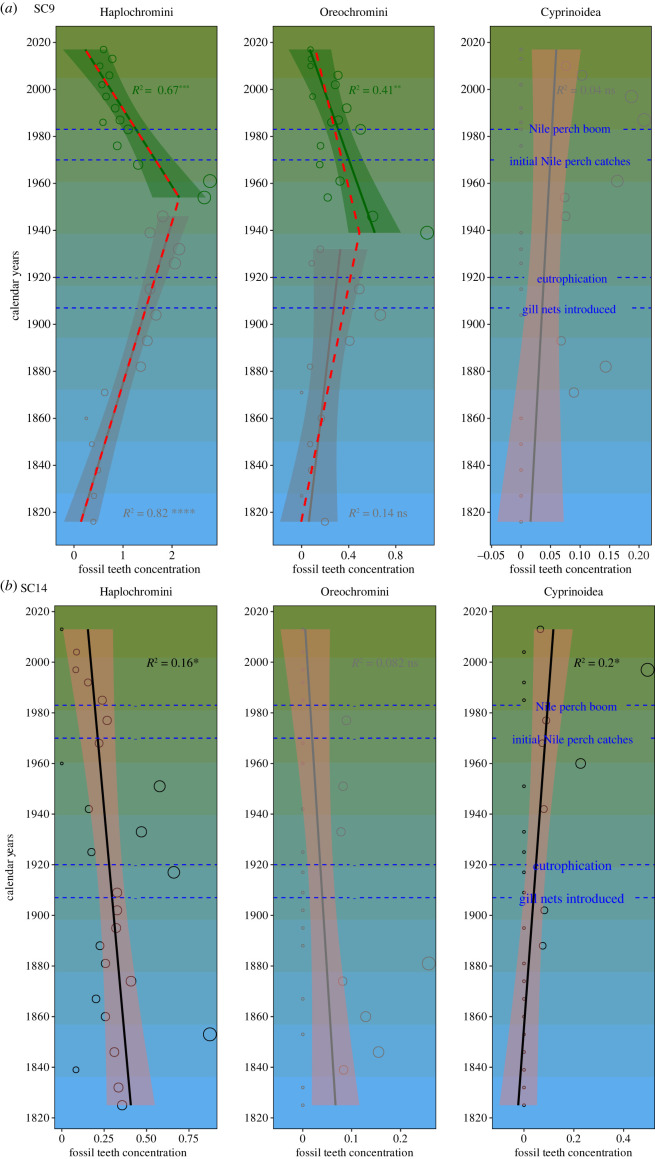
The fossil concentration (teeth per cm^3^) from (*a*) core SC9 (12.5 m water depth) and (*b*) core SC14 (14.5 m water depth; Mwanza Gulf, Lake Victoria). The red line is the estimated regression for the breakpoint analysis using the segmented package in R [30], with the peak indicating the breakpoint. The black, grey and green lines represent the linear models fitted to the data (black is without, grey is before and green is after the breakpoint). R^2^ of 0 to 1 denoting weak and strong correlation, respectively. The *p*-value shown as asterisks indicates statistical significance (* *p* < 0.05; n.s. *p* ≥ 0.05). The shaded band indicates the pointwise 95% confidence interval around the fitted lines. The blue lines mark the years with historical events including: intense fishing as the gillnets were introduced in 1907 [31], the introduction of beach seines and the onset of significant eutrophication in 1920 [32,33] (King *et al.,* [28]), the introduction of Nile perch and Nile tilapia in 1950 but only started appearing in Mwanza trawls in 1961, and the Nile perch boom in the Gulf in 1982/3 [34–36].

The time series reconstruction from the two cores reaches back approximately 200 years. A total of 635 fossil fish teeth were found and assigned to four major fish taxa Mochokidae, Haplochromini, Oreochromini and Cyprinoidea (electronic supplementary material, table S1, electronic supplementary material, SI*. 6*). In both cores, haplochromine cichlids' fossil teeth dominated the assemblage but the total abundance of fish was about fivefold higher in the more inshore site (SC9) (electronic supplementary material, figure S3, SI*. 6*), consistent with the general inshore–offshore productivity gradient [13]. In the deeper and more offshore site, SC14, the year–abundance relationship shows no significant change of slope for the detected breakpoint (electronic supplementary material, table S2), and no significant changes in the abundance of oreochromine cichlids were evident, the abundances were very low throughout the time series (figure 1*,* electronic supplementary material, figures S3 and S4). In comparison, haplochromine cichlids have been significantly decreasing since the early ninteenth century (figure 1). In SC9, the site nearer to the shore and in shallower waters, the year–abundance relationship shows a significant change of slope for the detected breakpoints in both cichlid groups. Oreochromine cichlids have a slight but non-significant increase and began to decline significantly after a breakpoint that is situated around approximately 1939 CE (±5 years) (figure 1). The haplochromines show a highly significant increase from the early ninteenth century until a highly significant change of slope for the breakpoint detected around approximately 1954 CE (±3 years), followed by a highly significant decrease (figure 1).

These data suggest that the stocks of these smaller cichlids that occupy the inshore and sublittoral environments in many different species began to decline in sublittoral waters from the 1800s and in more inshore waters in the 1950s. Records show the completion of the railway in 1901 connecting the lake shore to inland, but in Mwanza which is the closest port to our coring sites, the railway was completed in 1928 enabling wider access to the lake [31,37]. Generally, there exists a strong link between anthropogenic impacts and biodiversity loss [38], and haplochromine fish stocks in LV exhibit limited resilience when exposed to prolonged intensive exploitation or substantial predation pressure [39]. However, a direct effect of fishing is unlikely to explain the decline of smaller haplochromines because the application of the new fishing gear was largely confined to closer to the shore areas of the lake and used large mesh sizes (127 mm) to target larger fish [11], but failed to retain small haplochromines. On the other hand, the hypothesis that the main cause for the cichlid fish decline was the invasion of the Nile perch [5], is also not supported by the fossil evidence. Nile perch catches, and therefore likely abundances, only became prominent in the Mwanza Gulf by the 1980s. Our findings show the decline of cichlids before the introduction of Nile perch and are consistent with data from trawl surveys that suggested a decline in haplochromine catches preceded the Nile perch upsurge in 1982/3 but they could not resolve the timing of the decline [10,40]. Meanwhile, the human population in Mwanza doubled between 1975 and 2015 [41] and added to the anthropogenic eutrophication. The timing of the onset of the decline in haplochromine cichlids that we detect here followed after the measurable onset of eutrophication of the lake [32,33] and in Mwanza [28,32,33]. Seemingly, the timing of the decrease suggests the driver for the decline follows the progressive and detectable eutrophication that transformed the fish habitats and concurrent intensified fishing pressure.

The cyprinoid abundance trends in our data show no significant change of slope for the detected breakpoint in SC9, and no significant trends. Although, in SC14, there was also no significant year–abundance relationship change of slope (figure 1), there was a significant increasing trend from the early 1800s until the present (figure 1). Several fisheries reports suggested the native cyprinoid, *Rastrineobola argentea*, has been increasing and thriving in the lake since the 1980s [42,43]. The increase of *Rastrineobola* in the 1980s and early 1990s coincided with the temporal decline of zooplanktivorous haplochromine cichlids with which they had habitat overlap. It has been suggested that the cichlids’ decline may have reduced competitive pressures on *Rastrineobola*.

## 4. Conclusion

Previous knowledge about the changes in LV fish communities relied on a relatively limited 30–40 years time series of monitoring data from the Mwanza Gulf. Fish fossil records from sediment cores provide longer temporal records and offer a new fossil perspective on debates regarding the factors that led to the loss of cichlid fish abundance and species richness in LV. Our data show that haplochromine and oreochromine cichlids both started to decline before Nile perch was initially caught in the 1970s and long before it was caught in significant numbers in Mwanza Gulf in 1982/3. Intensified fishing and the measurable onset of eutrophication preceded the downward trends in cichlid abundances. Based on this, we suggest that lake eutrophication and associated ecosystem processes initiated the decline of the smaller haplochromines, and intensified fishing may have initiated the decline of larger cichlids, i.e. oreochromines and the largest haplochromine species. The Nile perch boom that began in the 1980s may have exacerbated these effects, speeding up the collapse of offshore haplochromine species richness. Our findings suggest conservation strategies should primarily be directed at mitigating eutrophication and promoting sustainable fishing.

## Data Availability

The fossil data are available from the Dryad Digital Repository: https://doi.org/10.5061/dryad.95x69p8rp [44]. Supplementary material is available online [45].

## References

[1] Johnson TC et al. 1996 Late Pleistocene desiccation of Lake Victoria and rapid evolution of cichlid fishes. Science **273**, 1091-1093. (10.1126/science.273.5278.1091) 8688092

[2] Seehausen O. 2002 Patterns in fish radiation are compatible with Pleistocene desiccation of Lake Victoria and 14 600 year history for its cichlid species flock. Proc. R. Soc. B **269**, 491-497. (10.1098/rspb.2001.1906) PMC1690916 11886641

[3] Verschuren D, Johnson TC, Kling HJ, Edgington DN, Leavitt PR, Brown ET, Talbot MR, Hecky RE. 2002 History and timing of human impact on Lake Victoria, East Africa. Proc. R. Soc. B **269**, 289-294. (10.1098/rspb.2001.1850) PMC1690894 11839198

[4] Marten GG. 1979 Impact of fishing on the inshore fishery of Lake Victoria (East Africa). J. Fish. Res. Board Can. **36**, 891-900. (10.1139/f79-127)

[5] Barel CDN et al. 1985 Destruction of fisheries in Africa's lakes. Nature **315**, 19-20. (10.1038/315019a0)

[6] Witte F, Goldschmidt T, Wanink J, van Oijen M, Goudswaard K, Witte-Maas E, Bouton N. 1992 The destruction of an endemic species flock: quantitative data on the decline of the haplochromine cichlids of Lake Victoria. Environ. Biol. Fishes **34**, 1-28. (10.1007/BF00004782)

[7] Witte F. 1980 Initial results of the ecological survey of the haplochromine cichlid fishes from the Mwanza Gulf of Lake Victoria (Tanzania): breeding patterns, trophic and species distribution. Netherlands J. Zool. **31**, 175-202. (10.1163/002829680X00230)

[8] Kaufman L. 1992 Catastrophic change in species-rich freshwater ecosystems. Bioscience **42**, 846-858. (10.2307/1312084)

[9] Seehausen O, Witte F, Katunzi EF, Smits J, Bouton N. 1997 Patterns of the remnant cichlid fauna in southern Lake Victoria: Patrones de la Fauna de Cíclidos Remanentes en el Sur del Lago Victoria. Conserv. Biol. **11**, 890-904. (10.1046/j.1523-1739.1997.95346.x)

[10] van Zwieten PAM, Kolding J, Plank MJ, Hecky RE, Bridgeman TB, MacIntyre S, Seehausen O, Silsbe GM. 2016 The Nile perch invasion in Lake Victoria: cause or consequence of the haplochromine decline? Can. J. Fish. Aquat. Sci. **73**, 622-643. (10.1139/cjfas-2015-0130)

[11] Graham M. 1929 The Victoria Nyanza and its fisheries: a report on the fishing survey of lake Victoria, 1927–1928, and appendices. London, UK: Crown Agents for the colonies.

[12] Kudhongania AW, Cordone AJ. 1974 Batho-spatial distribution pattern and biomass estimate of the major demersal fishes in Lake Victoria. Afr. J. Trop. Hydrobiol. Fish. **3**, 15-31.

[13] Witte F, Goldschmidt T, Wanink JH. 1995 Dynamics of the haplochromine cichlid fauna and other ecological changes in the Mwanza Gulf of Lake Victoria. In The impact of species changes in African lakes (eds TJ Pitcher, PJB Hart), pp. 83-110. London, UK: Chapman & Hall.

[14] Syanya FJ, Mathia WM, Winam ZO. 2024 Vanishing splendor: a comprehensive review of the decline in the original fish fauna of Lake Victoria. Mar. Fish. Sci. **37**, 209-231. (10.47193/mafis.3722024010507)

[15] Cuenca-Cambronero M et al. 2022 An integrative paleolimnological approach for studying evolutionary processes. Trends Ecol. Evol. **37**, 488-496. (10.1016/j.tree.2022.01.007) 35183376

[16] Cohen AS, Gergurich EL, Kraemer BM, McGlue MM, McIntyre PB, Russell JM, Simmons JD, Swarzenski PW. 2016 Climate warming reduces fish production and benthic habitat in Lake Tanganyika, one of the most biodiverse freshwater ecosystems. Proc. Natl Acad. Sci. USA **113**, 9563-9568. (10.1073/pnas.1603237113) 27503877 PMC5003268

[17] Navarro N, Montuire S, Laffont R, Steimetz E, Onofrei C, Royer A. 2018 Identifying past remains of morphologically similar vole species using molar shapes. Quaternary **1**, 20. (10.3390/quat1030020)

[18] Muschick M et al. 2018 Arrival order and release from competition does not explain why haplochromine cichlids radiated in Lake Victoria. Proc. R. Soc. B **285**, 20180462. (10.1098/rspb.2018.0462) PMC5966608 29743255

[19] Ngoepe N et al. 2023 A continuous fish fossil record reveals key insights into adaptive radiation. Nature **622**, 315-320. (10.1038/s41586-023-06603-6) 37794187 PMC10567567

[20] Renberg I, Hansson H. 2008 The HTH sediment corer. J. Paleolimnol. **40**, 655-659. (10.1007/s10933-007-9188-9)

[21] Witte F, Wanink JH, Kishe-Machumu M, Mkumbo OC, Goudswaard PC, Seehausen O. 2007 Differential decline and recovery of haplochromine trophic groups in the Mwanza Gulf of Lake Victoria. Aquat. Ecosyst. Health Manage. **10**, 416-433. (10.1080/14634980701709410)

[22] Fryer G, Iles TD. 1972 The cichlid fishes of the Great Lakes of Africa: their biology and evolution, vol. 641. Edinburgh, UK: Oliver and Boyd.

[23] Greenwood PH. 1974 Cichlid fishes of Lake Victoria, East Africa: the biology and evolution of a species flock. Bull. British Mus. Nat. Hist. (Zool.), (Suppl. 6), 1-134.

[24] Witte F, Van Oijen MJP. 1990 Taxonomy, ecology and fishery of Lake Victoria haplochromine trophic groups. Zoologische Verhandelingen **262**, 1-47.

[25] Seehausen O. 1996 Lake Victoria rock cichlids: taxonomy, ecology and distribution. Zevenhuizen, The Netherlands: Verduyn Cichlids.

[26] Winfield IJ, Nelson JS. 2012 Cyprinid fishes: systematics, biology and exploitation. London, UK: Springer Science & Business Media.

[27] Alsafy MAM, Bassuoni NF, Hanafy BG. 2018 Gross morphology and scanning electron microscopy of the *Bagrus bayad* (Forskal, 1775) oropharyngeal cavity with emphasis to teeth–food adaptation. Microsc. Res. Tech. **81**, 878-886. (10.1002/jemt.23050) 29737577

[28] King L et al. In preparation. Anthropogenic eutrophication drives major food web changes in Mwanza Gulf, Lake Victoria. 10.1007/s10021-024-00908-x PMC11182866 38899133

[29] Muggeo VMR. 2008 Segmented: an R package to fit regression models with broken-line relationships. R News **8**, 20-25.

[30] Muggeo VMR, Muggeo MVMR. 2017 Package ‘segmented.’ Biometrika **58**, 516.

[31] Garrod DJ. 1961 The history of the fishing industry of Lake Victoria, East Africa, in relation to the expansion of marketing facilities. East Afri. Agric. For. J. **27**, 95-99. (10.1080/00128325.1961.11661759)

[32] Hecky RE. 1993 The eutrophication of Lake Victoria. SIL Proceedings, 1922-2010 **25**, 39-48. (10.1080/03680770.1992.11900057)

[33] Sitoki L, Gichuki J, Ezekiel C, Wanda F, Mkumbo OC, Marshall BE. 2010 The environment of Lake Victoria (East Africa): current status and historical changes. Int. Rev. Hydrobiol. **95**, 209-223. (10.1002/iroh.201011226)

[34] Okemwa EN. 1984 Potential fishery of Nile perch *Lates niloticus* Linne (Pisces: Centropomidae) in Nyanza Gulf of Lake Victoria, East Africa. Hydrobiologia **108**, 121-126. (10.1007/BF00014871)

[35] Frans Witte KG. 1997 The catfish fauna of Lake Victoria after the Nile perch upsurge. Environ. Biol. Fishes **49**, 21-43. (10.1023/A:1007311708377)

[36] Taabu-Munyaho A, Marshall BE, Tomasson T, Marteinsdottir G. 2016 Nile perch and the transformation of Lake Victoria. Afr. J. Aquat. Sci. **41**, 127-142. (10.2989/16085914.2016.1157058)

[37] Ogonda RT. 1992 Transport and communications in the colonial economy. In An economic history of Kenya (eds WR Ocheing', RM Maxon). Nairobi, Kenya: East African Educational Publishers Ltd.

[38] Isbell F et al. 2017 Linking the influence and dependence of people on biodiversity across scales. Nature **546**, 65-72. (10.1038/nature22899) 28569811 PMC5460751

[39] Witte F, Goudswaard PC. 1985. Aspects of the Haplochromine fishery in southern Lake Victoria. Annex 1.5. FAO Fisheries Report. no. 335. Rome, Italy: FAO.

[40] Kolding J, Medard M, Mkumbo O, Van Zwieten PAM. 2014 Status, trends and management of the Lake Victoria fisheries. Inland fisheries evolution and management—case studies from four continents. FAO Fisheries and Aquaculture Technical Paper no. 579. Rome, Italy: FAO.

[41] Worrall L, Colenbrander S, Palmer I, Makene F, Mushi D, Mwijage J, Martine M, Godfrey N. 2017 Better urban growth in Tanzania: preliminary exploration of the opportunities and challenges. Coalition for Urban Transitions. See http://newclimateeconomy.net/content/cities-working-papers.

[42] Bwathondi POJ. 1990 *The state of Lake Victoria fisheries, Tanzanian sector.* National Report presented to the Fifth Session of the CIFA Sub-Committee for the Development and Management of the Fisheries of Lake Victoria. Mwanza, Tanzania, 12–14 Sept. 1989. Rome, Italy: FAO.

[43] Kudhongania AW, Twongo T, Ogutu-Ohwayo G. 1992 Impact of the Nile perch on the fisheries of Lakes Victoria and Kyoga. Hydrobiologia **232**, 1-10. (10.1007/BF00014605)

[44] Ngoepe N et al. 2024 Data from: Testing alternative hypotheses for the decline of cichlid fish in Lake Victoria using fish fossils time series from sediment cores. Dryad Digital Repository. (10.5061/dryad.95x69p8rp) PMC10950456 38503343

[45] Ngoepe N et al. 2024 Testing alternative hypotheses for the decline of cichlid fish in Lake Victoria using fish fossils time series from sediment cores. Figshare. (10.6084/m9.figshare.c.7098718) PMC10950456 38503343

